# Ataxia and Encephalitis in a Young Adult with EBV Mononucleosis: A Case Report 

**DOI:** 10.1155/2013/516325

**Published:** 2013-05-28

**Authors:** Rashid S. Hussain, Naaz A. Hussain

**Affiliations:** ^1^Warren Alpert Medical School of Brown University, Providence, RI 02912, USA; ^2^Johns Hopkins Community Physicians, Frederick, MD 21701, USA

## Abstract

Neurological manifestations of mononucleosis are extremely rare, occurring in about 1% of all cases. However, when they occur, appropriate treatment must be undertaken to ensure appropriate symptomatic management and reduce morbidity. We present the case of a 25-year-old graduate student with weeklong complaints of fever, sore throat, fatigue, nausea, and “dizziness.” She later developed increased sleep requirements, ataxia, vertigo, and nystagmus with a positive EBV IgM titer confirming acute infectious mononucleosis. The patient was clinically diagnosed with EBV-associated cerebellitis and encephalitis, displaying neurological and psychiatric impairment commonly seen in postconcussion syndrome. MRI showed no acute changes. She was started on valacyclovir and a prednisone taper, recovering by the end of twelve weeks. Though corticosteroids and acyclovir are not recommended therapy in patients presenting with EBV-associated ataxia, clinicians may want to keep a low threshold to start these medications in case more serious neurological sequelae develop.

## 1. Introduction

Mononucleosis is a viral illness which classically presents in young adults as a triad of fatigue, pharyngitis, and lymphadenopathy. In most cases, it has an insidious onset over 1-2 weeks, sometimes associated with thrombocytopenia and transaminitis, with gradual self-resolution [1]. However, serious neurological manifestations including seizures, ataxia, meningitis, encephalitis, transverse myelitis, Guillain-Barre syndrome, autonomic dysfunction, anxiety, and depression can occur in about 1% of cases [1–3]. These have been documented predominantly in the pediatric literature as part of a postviral syndrome [1, 2, 4, 5]. The patient in this case was older than the usual cohort and presented with early neurological complications including ataxia with later exacerbation of vertigo, difficulty in concentrating, noise sensitivity, and mental fatigue from minor tasks likely due to encephalitis with clinical presentation similar to postconcussion syndrome [3, 6]. This was debilitating for the patient who deteriorated from an independent graduate student to being home-bound and dependent for all IADLs. Neurological complications of mononucleosis need to be considered with the diagnosis to ensure timely symptom management and appropriate assurance for the patient. 

## 2. Case Presentation

A 25-year-old female graduate student presented to her Student Health Services Office with weeklong complaints of fever, sore throat, fatigue, nausea, and “dizziness.” She denied vertigo, history of migraines, recent trauma, drug/alcohol use, or exposure to ticks. Initial evaluation revealed negative rapid strep and negative monospot. Patient was diagnosed with a viral infection and discharged back to her dorm. After discharge, she had exacerbating nausea with emesis, vertigo, tension headache, mental fatigue, gait unsteadiness, and increased sleep requirements. She was escorted by a friend to the clinic four days later and had labs drawn. Patient was prescribed meclizine and was told to take an OTC dextrose/fructose/phosphoric acid solution for symptom management.

Because the patient's condition continued to deteriorate, she decided to fly home. Upon arrival at the airport, her mother, a physician, noticed she was clearly ataxic. She was reevaluated at the Johns Hopkins Community Physicians' Office and was found to be afebrile with no evidence of rashes, though she presented with bilateral nystagmus, cervical lymphadenopathy, exudative pharyngitis, and dysarthria but no neck stiffness. She was alert and oriented and had CN II-XII intact other than as noted. Sensory and motor exam were within normal limits. Patient had symmetric reflexes, normal heel-to-shin and finger-to-nose, and rapid alternating movements intact, but a positive Romberg and a wide-based ataxic gait. Remainder of physical exam was noncontributory. Labs had returned by this point and were found to be EBV VCA-IgM positive and EBV VCA-IgG negative, with atypical lymphocytes consistent with early mononucleosis. Heterophile Ab and rapid strep tests remained negative. Throat cultures were confirmed to be negative. Repeat labs were drawn confirming the previous. Protein electrophoresis revealed IgG polyclonal hypergammaglobulinemia. Patient had also developed transaminitis with AST 31 ALT 59 and lymphocytosis of  55%. MRI showed no evidence of signal intensity or mass effect. A Neurological consult was obtained which agreed with the diagnosis of viral cerebellitis with possible vestibular neuronitis, and the patient was discharged home with services for physical therapy. After an informal consult with Infectious Disease, due to the atypical and complicated course of EBV, the patient was started on a ten-day course of valacyclovir along with a prednisone taper. Lumbar puncture was not conducted.

Patient conducted home physical and speech therapy for 4–6 weeks. Initially, the patient was extremely worried about the possible permanence of any symptoms as she was well aware of her symptoms and their potential long-term impact on her personal and professional life. Processing speed was delayed, and patient was distressed upon learning she was reading at a 6th grade level. Patient was unable to type and had illegible handwriting. A formal neuropsychological evaluation was not conducted. With time and spiritual and emotional support, patient was able to regain resolve. Constitutional symptoms resolved soon after coming home, and sleep requirements recovered to baseline over the course of two weeks. Patient gradually regained functionality with IADLs with loss of ataxic symptoms. She was able to return to baseline by twelve weeks. 

## 3. Discussion 

The case presented here of ataxia, cerebellitis, and encephalitis following EBV infection in a young adult is a rare occurrence. To date, ataxia secondary to either mononucleosis or postinfection syndrome has been largely identified in pediatric case reports [1, 2, 4, 5]. It is a rare symptomatology in young adults, especially in the context of a clinical picture including symptoms of an acute, likely primary, viral encephalitis [3, 7]. The differential for acute ataxia in this population includes cerebellar abscess, head trauma, vertebrobasilar dissection, alcohol toxicity, tick paralysis, seizure, and acute demyelinating encephalomyelitis. Other infectious causes include *Listeria monocytogenes, Borrelia burgdorferi,* HIV, coxsackie, EEE, and herpes viruses [4]. These were unlikely given the aforementioned history, labs, and normal MRI. 

Nonetheless, the encephalitic symptoms of this patient could have been minimized if treatment for EBV and inflammation were started earlier. The current literature suggests that corticosteroids do not necessarily provide benefit to patients with ataxia after EBV infection [2, 4]. This is thought to apply largely for postviral ataxia and was the course initially followed. For encephalitis, however, it is documented that corticosteroid use can improve clinical outcomes by reducing inflammation and cranial pressure [8, 9]. Though the evidence for corticosteroid use is more strongly documented for postviral encephalitis and acute demyelinating encephalomyelitis, it can be used for primary viral encephalitis as well [9]. Additionally, earlier treatment could have begun with acyclovir, which has been recommended for CNS involvement with EBV [8, 10]. 

## 4. Conclusion

It is important that cases of EBV with encephalitis, cerebellitis, and ataxia be documented and treated early to prevent progression of illness and any possible residual effects. Though the treatment of EBV with ataxia does not necessitate corticosteroid or acyclovir treatment, physicians should keep a low threshold for their use due to the risk of more severe CNS involvement such as encephalitis where such therapy is beneficial. In this case, early intervention could have decreased the severity and duration of illness, thus improving the patient's quality of life. As this patient had a severe loss of function and depressive symptoms because of her illness, this would have helped both her to confront her illness faster. 
